# Hesperidin Attenuates Mercury Chloride-Induced Neurotoxicity in Rats by Modulating Oxidative Stress, Neuroinflammation, Apoptosis, Autophagy and ER Stress

**DOI:** 10.1007/s12011-026-04987-w

**Published:** 2026-02-12

**Authors:** Samet Tekin, Merve Bolat, Ismail Bolat, Ömercan Alat, Burak Batuhan Laçin, Burak Çınar, Tuba Karaarslan, Mehmet Emin Kanat, Furkan Aykurt, Emin Şengül, Mohamad Warda, Serkan Yıldırım

**Affiliations:** 1 Department of Physiology, Faculty of Veterinary Medicine, Atatürk University, Erzurum, Türkiye; 2 Department of Pathology, Faculty of Veterinary Medicine, Atatürk University, Erzurum, Türkiye; 3 Department of Biochemistry, Faculty of Veterinary Medicine, Atatürk University, Erzurum , Türkiye; 4 Department of Biochemistry, Faculty of Veterinary Medicine, Cairo University, Giza, Egypt; 5 Department of Medical Pharmacology, Faculty of Medicine, Atatürk University, Erzurum, Türkiye; 6 Department of Pathology, Faculty of Veterinary Medicine, Kyrgyzstan-Turkey Manas University, Bishkek, Kyrgyzstan

**Keywords:** Apoptosis, Autophagy, Endoplasmic reticulum stress, Hesperidin, Inflammation, Mercury chloride

## Abstract

Mercury chloride (HgCl₂) is a well-known environmental toxicant that can induce neurotoxicity through oxidative stress, neuroinflammation, endoplasmic reticulum (ER) stress, dysregulated autophagy, and apoptosis. This study evaluated the potential neuroprotective effects of hesperidin (HES), a bioactive flavonoid with antioxidant and anti-inflammatory properties, against HgCl₂-induced brain injury in rats. Sixty male Sprague Dawley rats received 1.23 mg/kg HgCl₂ intraperitoneally for 7 days, while HES was administered orally at doses of 100, 200, or 400 mg/kg. HgCl₂ exposure resulted in elevated lipid peroxidation, impaired antioxidant status, increased pro-inflammatory cytokines (TNF-α, IL-1β, IL-6), and reduced IL-10 levels. Upregulation of Bax and caspase-3, downregulation of Bcl-2 and BDNF, along with increased GFAP immunoreactivity, indicated enhanced neuronal apoptosis and astrocyte activation. Furthermore, increased Beclin-1, LC3A/B, and ER stress-related markers (GRP78, PERK, ATF4, XBP1, IRE1, CHOP) suggested disturbances in cellular homeostasis. HES treatment—most notably at 400 mg/kg—attenuated oxidative stress, improved antioxidant enzyme activities, reduced pro-inflammatory responses while partially restoring IL-10, and modulated apoptosis, autophagy, and ER stress-associated pathways. In addition, increased BDNF levels following HES administration may indicate improved neuronal plasticity. Collectively, these findings suggest that hesperidin may have therapeutic potential as a neuroprotective agent against HgCl₂-induced neurotoxicity by modulating multiple molecular pathways involved in oxidative damage, inflammation, apoptosis, autophagy, and ER stress.

## Introduction

Mercury and its compounds have long remained a critical topic of investigation in the fields of environmental toxicology and neuroscience. Among the various forms of mercury—elemental mercury, inorganic mercury salts, and organic mercury compounds—the inorganic compound mercury chloride (HgCl₂) is of particular concern due to its tendency to accumulate in biological tissues and its long half-life, making it a significant environmental and public health threat [1, 2]. Although mercury exposure can affect multiple organ systems, the central nervous system is one of the most vulnerable targets because both organic and inorganic forms are capable of crossing the blood–brain barrier. This vulnerability is supported by evidence showing that HgCl₂ exposure leads to irreversible neurological outcomes, including impairments in motor coordination, learning, and memory [3, 4, 5, 6, 7].

At the molecular level, HgCl₂-induced neurotoxicity is known to progress through multiple interconnected mechanisms. Oxidative stress, driven by excessive production of reactive oxygen species, represents one of the earliest and most prominent outcomes of exposure, leading to damage in lipids, proteins, and DNA. Inflammatory pathways—characterized by microglial activation and NF-κB-mediated cytokine release—further exacerbate this oxidative disruption, ultimately compromising synaptic integrity [8]. Apoptosis is also a key component of this mechanism, with studies reporting activation of mitochondrial pathways characterized by increased Bax and caspase-3 expression and a corresponding decrease in Bcl-2 levels [9]. In addition, endoplasmic reticulum (ER) stress triggered by the accumulation of misfolded proteins is characterized by increased expression of markers such as GRP78, PERK, ATF4, XBP1, IRE1, and CHOP, and when prolonged, this response accelerates cellular death [10, 11]. Taken together, these findings indicate that HgCl₂ contributes to neurodegeneration through an integrated toxicity network involving oxidative stress, inflammation, apoptosis, and ER stress.

In recent years, the therapeutic potential of natural antioxidant compounds against heavy metal toxicity has gained increasing attention. Among these compounds, the flavonoid hesperidin stands out for its strong free radical–scavenging capacity, its ability to reduce oxidative damage, support endogenous antioxidant defenses, and modulate inflammatory responses [12]. Furthermore, studies in various experimental models have reported that hesperidin suppresses inflammation and apoptosis while providing both structural and functional protection to neural tissue [13]. Beyond these effects, accumulating evidence indicates that hesperidin can also regulate intracellular stress responses by modulating endoplasmic reticulum (ER) stress and autophagy-related signaling pathways, particularly under conditions of toxic insult [14, 15]. In this context, hesperidin emerges as a promising protective agent against HgCl₂-induced neurotoxicity.

In light of this evidence, the present study aimed to investigate the protective effects of hesperidin on the multifaceted neurotoxic mechanisms triggered by HgCl₂ exposure, including oxidative stress, inflammation, apoptosis, autophagy, and ER stress.

## Materials and Methods

### Chemicals

Mercury chloride (HgCl₂; CAS No: 7487-94-7) and hesperidin (CAS No: 520-26-3, ≥ 80% (HPLC)) used in this study were obtained from Sigma-Aldrich (St. Louis, MO, USA). The ELISA kits employed in the experiments were supplied by YL-Biont (Shanghai, China) and included the Rat Malondialdehyde (MDA) ELISA Kit (YLA0029RA), Rat Superoxide Dismutase (SOD) ELISA Kit (YLA0115RA), Rat Glutathione (GSH) ELISA Kit (YLA1511RA), Rat Interleukin-1β (IL-1β) ELISA Kit (YLA0030RA), Rat Interleukin-6 (IL-6) ELISA Kit (YLA0031RA), Rat Interleukin-10 (IL-10) ELISA Kit (YLA0440RA), and Rat Tumor Necrosis Factor-α (TNF-α) ELISA Kit (YLA0118RA). All analyses were performed in accordance with the manufacturers’ instructions. All ELISA-derived results were normalized to tissue weight and expressed as nanograms per gram of tissue (ng/g tissue).

## Groups and Experimental Procedure

Sixty male Sprague Dawley rats (220–250 g, 12 weeks old) used in this study were obtained from the Atatürk University Medical Experimental Research Center (ATADEM, Erzurum, Turkey). Following a 7-day acclimatization period, animals were housed in bedding-lined cages enriched with environmental stimulation materials. Throughout the experiment, temperature was maintained at 25 ± 2 °C with 55 ± 10% relative humidity under a 12-h light/dark cycle, and food and water were provided ad libitum. Rats were allocated to experimental groups using a computer-based simple randomization method, and all procedures were performed at the same time of day to minimize circadian variability. The experimental protocol was approved by the Atatürk University Animal Experiments Ethics Committee (Approval No: 2024/13/299) and conducted in accordance with ethical guidelines. Hesperidin was prepared as a homogeneous suspension in distilled water and administered intragastrically (1 mL/kg). The suspension was freshly prepared each day and thoroughly vortexed immediately before administration to ensure uniform distribution and accurate dosing.

The dose of HgCl₂ (1.23 mg/kg, intraperitoneal) was selected based on previous studies demonstrating consistent induction of neurotoxicity without causing excessive mortality [16]. The selected hesperidin doses (100, 200, and 400 mg/kg, oral) were chosen according to earlier experimental studies reporting dose-dependent antioxidant and neuroprotective effects within this range, while maintaining a favorable safety profile [17]. This dosing strategy allowed evaluation of both the toxic effects of HgCl₂ and the potential dose-dependent protective effects of hesperidin.

HgCl₂ was administered intraperitoneally to ensure consistent systemic exposure and reliable induction of neurotoxicity, as commonly applied in experimental mercury toxicity models. In contrast, hesperidin was administered orally to reflect its natural route of intake as a dietary flavonoid and to better mimic its potential translational use.

### Control

Rats received 1 mL distilled water orally once daily for 7 days.

### HES400

Rats received hesperidin at 400 mg/kg orally once daily for 7 days.

### HgCl₂

Rats were administered HgCl₂ at 1.23 mg/kg intraperitoneally once daily for 7 days [16].

### HgCl₂ + HES100

Rats received HgCl₂ (1.23 mg/kg, i.p.) followed 30 min later by hesperidin (100 mg/kg, oral) once daily for 7 days [17].

### HgCl₂ + HES200

Rats received HgCl₂ (1.23 mg/kg, i.p.) followed 30 min later by hesperidin (200 mg/kg, oral) once daily for 7 days [17].

### HgCl₂ + HES400

Rats received HgCl₂ (1.23 mg/kg, i.p.) followed 30 min later by hesperidin (400 mg/kg, oral) once daily for 7 days [17].

All behavioral assessments were performed after completion of the 7-day treatment period and prior to euthanasia. Behavioral tests were conducted following the final drug administration, and animals were sacrificed 24 h after the completion of behavioral testing for tissue collection. All behavioral assessments were performed on the same day by an investigator blinded to the experimental groups.

## Behavioral Tests

### Open Field Test (OFT)

This test was used to evaluate locomotor activity and anxiety-like behavior. The open field apparatus consisted of a square arena (42 × 42 × 42 cm) equipped with an infrared-based tracking system (MAY ACT 508, Commat, Ankara, Türkiye). No external light source was used during testing; locomotor parameters were detected exclusively through infrared sensors to prevent light-induced variations in activity. Each rat was gently placed in the center of the arena and allowed to explore freely for 10 min. Total distance traveled, immobility duration, and rearing activity were automatically detected and quantified. After each trial, the arena was thoroughly cleaned with a non-scented disinfectant to eliminate any odors, fecal traces, or urine residues that could influence the subsequent animal’s behavior.

### Elevated Plus Maze (EPM)

Anxiety-like behavior was further evaluated using the EPM test. The maze consisted of two open arms and two closed arms, each 10 cm wide and 1 m in length, intersecting at a central platform elevated above the floor. After each test session, the apparatus was cleaned to prevent odor-related bias. The time spent in open and closed arms, as well as the number of entries into open arms, was recorded for 5 min using EthoVision XT 10.0 video-tracking software.

### Tissue Collection

Twenty-four hours after the final administration, all rats were sacrificed under moderate sevoflurane anesthesia (Sevorane^®^; Queenborough, UK), and cerebral cortex tissues were collected for biochemical and molecular analyses. A portion of the cerebral cortex tissue was gently rinsed with physiological saline and fixed in 10% neutral buffered formalin for histopathological and immunohistochemical examinations. The remaining cerebral cortex tissues were again rinsed with physiological saline, rapidly frozen in liquid nitrogen, and stored at − 80 °C until further biochemical and molecular analyses were performed.

### Biochemical Analyses

Oxidative, antioxidant, and inflammatory parameters—including malondialdehyde (MDA), superoxide dismutase (SOD), glutathione (GSH), and the cytokines TNF-α, IL-6, IL-1β, and IL-10—were quantified spectrophotometrically using a BioTek EPOCH2 microplate reader at 450 nm, in accordance with the manufacturer’s instructions for the assay kits used. All biochemical measurements were normalized to gram of tissue to ensure accurate comparison across samples.

### Histopathological Examinations

Cerebral cortex tissue samples collected at the end of the experiment were fixed in 10% neutral buffered formalin for 48 h and subsequently processed through routine tissue preparation procedures before being embedded in paraffin blocks. Sections of 4 μm thickness were obtained from each block, stained with hematoxylin–eosin (H&E), and examined under a light microscope (Leica, Flexacam i5). Histopathological evaluation focused on detecting neuronal degeneration and necrosis in the cerebral cortex. Observed alterations were graded semi-quantitatively according to severity as absent (−), mild (+), moderate (++), or severe (+++) [18].

### Immunohistochemical Examinations

For immunohistochemical analyses, tissue sections mounted on adhesive (poly-L-lysine–coated) slides following routine tissue processing were deparaffinized and rehydrated. Endogenous peroxidase activity was blocked by incubating the sections in 3% H₂O₂ for 10 min. Antigen retrieval was then performed by heating the tissues in 1% TRIS-EDTA buffer (pH 6.1, 100X), followed by cooling to room temperature. To prevent nonspecific background staining, the sections were incubated with a protein block for 5 min. Subsequently, primary antibodies—BDNF (Cat. No: DF6387, dilution 1:100, Affinity) and GFAP (Cat. No: ab7260, dilution 1:100, Abcam)—were applied and incubated according to the manufacturers’ instructions. After the application of secondary antibodies, 3-amino-9-ethylcarbazole (AEC) chromogen was applied for visualization. Stained sections were examined under a light microscope (Leica, Flexacam i5). Following microscopic evaluation, ImageJ software was used to quantify immunopositive staining intensity [19].

### Immunofluorescence Examinations

For immunofluorescence analysis, tissue sections mounted on adhesive (poly-L-lysine–coated) slides were deparaffinized and rehydrated. Antigen retrieval was performed by heating the sections in 1% citrate buffer (pH 6.1, 100X), followed by cooling to room temperature. To reduce nonspecific background staining, the sections were incubated with a protein block for 5 min. Primary antibodies—BAX (Cat. No: sc-7480, mouse monoclonal, 1:100), Bcl-2 (Cat. No: sc-7382, mouse monoclonal, 1:100), and Caspase-3 (Cat. No: sc-56053, mouse monoclonal, 1:100)—were then applied and incubated according to the manufacturers’ protocols. After primary incubation, a fluorescent secondary antibody, fluorescein isothiocyanate (FITC; Cat. No: sc-2359, mouse monoclonal, 1:1000), was applied and the sections were kept in the dark for 45 min. Subsequently, mounting medium containing 4′,6-diamidino-2-phenylindole (DAPI; Cat. No: ab104140, 1:200) was added, and the sections were incubated for 5 min in the dark before coverslipping. The stained tissues were examined under a fluorescence microscope equipped with appropriate fluorescence attachments (Zeiss AXIO, Germany) [20].

### Western Blot Analyses

Western blot analysis was conducted to determine protein expression levels in cerebral cortex tissues. Approximately 50 mg of cerebral cortex tissue was homogenized in RIPA lysis buffer supplemented with a protease inhibitor cocktail (Santa Cruz Biotechnology, sc-24948, USA), followed by centrifugation at 16,000 g for 20 min at + 4 °C to obtain the supernatants. Total protein concentrations were quantified using the BCA Protein Assay Kit (Thermo Scientific, Pierce BCA, 23225, USA) based on a BSA standard curve. Equal amounts of protein were separated by SDS-PAGE using acrylamide/bis-acrylamide solution (Bio-Rad, 161 − 0156, USA) under reducing conditions. Proteins were transferred onto PVDF membranes (Bio-Rad, 162–0177, USA) using a wet transfer system, after which membranes were blocked in TBST containing 5% non-fat dry milk. Membranes were then incubated overnight at + 4 °C with primary antibodies targeting GRP78 (≈ 55–90 kDa, sc-13539), IRE1α (≈ 90–132 kDa, sc-390960), XBP1 (≈ 20–37 kDa, sc-8015), PERK (≈ 130–200 kDa, sc-377400), CHOP (≈ 23–34 kDa, sc-7351), ATF4 (≈ 45–55 kDa, sc-390063), and β-Tubulin (≈ 45–55 kDa, sc-5274). β-Tubulin served as the internal loading control.

Following primary incubation, membranes were washed and incubated with HRP-conjugated Goat anti-Mouse IgG-HRP secondary antibody (1:1000, sc-2005, Santa Cruz Biotechnology, USA). Protein bands were visualized using Clarity™ Western ECL Substrate (Bio-Rad, 170–5061, USA) and imaged using the ChemiDoc™ Imaging System (Bio-Rad, USA). Densitometric quantification of band intensities was performed in Image Lab 6.1 software, and target protein expression levels were normalized to β-Tubulin.

### qRT-PCR Analyses

Primers required to quantify the mRNA transcript levels of Beclin-1, LC3A, and LC3B genes were designed using the Primer-BLAST tool (Table 1).

**Table 1:** RT-PCR primer sequences

Gene	Sequences (5’-3’)	Accession No
LC3A	F: GACCATGTTAACATGAGCGA R: CCTGTTCATAGATGTCAGCG	NM_199500.2
LC3B	F: GAGCTTCGAACAAAGAGTGG R: CGCTCATATTCACGTGATCA	NM_022867.2
Beclin-1	F: TCTCGTCAAGGCGTCACTTC R: CCATTCTTTAGGCCCCGACG	NM_053739.2
B-actin	F: CAGCCTTCCTTCCTGGGTATG R: AGCTCAGTAACAGTCCGCCT	NM_031144.3

Total RNA was extracted from 100 mg of cerebral cortex tissue using QIAzol Reagent (QIAGEN, USA). Tissue samples were homogenized in 1 mL of QIAzol, followed by phase separation with 200 µL chloroform and centrifugation at 12,000 × g for 20 min at 4 °C. The upper aqueous phase was mixed with 500 µL isopropanol and centrifuged at 12,000 × g for 10 min to precipitate RNA. The resulting pellet was washed with 75% ethanol, recentrifuged at 7,500 × g for 5 min, air-dried, and dissolved in RNase-free DEPC-treated water. RNA concentration and purity were determined spectrophotometrically at 260/280 nm, and samples with A260/280 ratios between 1.8 and 2.2 were used for subsequent analyses.

To eliminate genomic DNA contamination, total RNA was treated with DNase I (Thermo Scientific, USA). A total of 1 µg RNA per sample was reverse-transcribed into cDNA using the miScript Reverse Transcription Kit (QIAGEN, USA) according to the manufacturer’s instructions. Synthesized cDNA was quantified spectrophotometrically, diluted to equal working concentrations, and used for quantitative real-time PCR.

qRT-PCR was performed using a QIAGEN Real-Time PCR System with SYBR Green 2X Rox Master Mix (QIAGEN, USA). Each reaction was carried out in triplicate and included gene-specific primers (Beclin-1, LC3A, LC3B) together with β-actin as the housekeeping reference gene. The thermal cycling conditions consisted of an initial activation at 95 °C for 10 min, followed by 40 cycles of denaturation at 95 °C for 15 s, annealing at 60 °C for 30 s, and extension at 72 °C for 30 s. Melt-curve analysis was performed to verify amplification specificity (Table 1).

Gene expression levels were calculated using the 2^^−ΔΔCt^ method after normalization to β-actin [21].

### Statistical Analysis

All data were expressed as mean ± standard deviation (SD). The normality of data distribution was assessed using the Shapiro–Wilk test. For normally distributed variables (biochemical, behavioral, immunohistochemical, and immunofluorescence measurements), group differences were analyzed using one-way analysis of variance (one-way ANOVA) followed by Tukey’s post hoc test. Non-normally distributed data, including H&E histopathological scores, were evaluated using the Kruskal–Wallis test with Dunn’s multiple comparison test. To minimize type I error related to multiple testing across numerous outcome variables, the false discovery rate (FDR) approach (Benjamini–Hochberg procedure) was applied where appropriate. All statistical analyses were performed using GraphPad Prism 10.6.1 (GraphPad Software, San Diego, CA, USA), and *p* < 0.05 was considered statistically significant.

### Effects of Hesperidin on Behavioral Outcomes in Mercury Chloride-Induced Neurotoxicity

In this study, mercury chloride was found to increase anxiety-like behaviors and impair locomotor activity in rats, while hesperidin—particularly at lower doses—significantly ameliorated these adverse effects. Findings from the Elevated Plus Maze and locomotor activity assessments demonstrate the neuroprotective potential of hesperidin, indicating that it may exert protective effects against HgCl₂-induced anxiety and motor behavior disturbances.

### Elevated Plus Maze Test

**Fig. 1:**
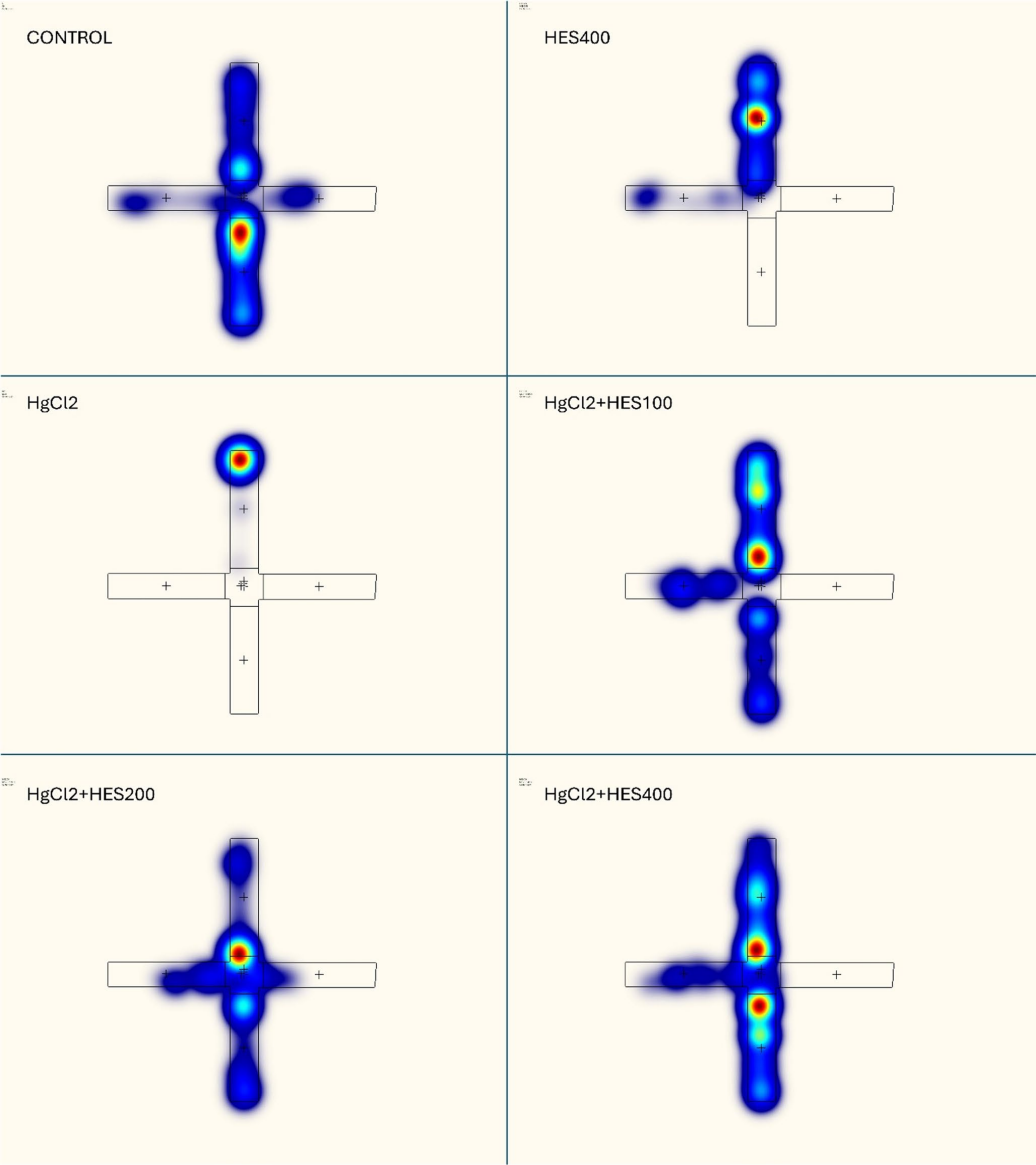
Representative heat map for each group in the Elevated Plus Maze test

The Elevated Plus Maze (EPM) test results demonstrated that mercury chloride (HgCl₂)-induced brain injury increased anxiety-like behavior, while hesperidin exerted a partially protective effect against these alterations. According to the graphical data, the HgCl₂ group spent a significantly longer duration in the closed arms, indicating heightened anxiety levels. Statistically, the HgCl₂ group spent more time in the closed area compared to the control group (*p* < 0.05). Moreover, a significant difference was observed between the HgCl₂ and HgCl₂+HES100 groups (*p* < 0.01), revealing that low-dose hesperidin (100 mg/kg) substantially reduced HgCl₂-induced anxiety-like behavior. Although the HgCl₂+HES200 and HgCl₂+HES400 groups also showed reduced closed-arm time compared to the HgCl₂ group, these differences were not statistically significant (ns, Fig. 1).

This finding suggests that the 200 and 400 mg/kg doses of hesperidin do not exert a marked restorative effect, whereas the 100 mg/kg dose may provide a stronger protective action. On the other hand, when HES400 was administered alone, it did not produce a significant difference compared with the control group (ns), indicating that hesperidin by itself does not alter anxiety-like behavior. Overall, in the EPM test, HgCl₂ administration increased anxiety, while hesperidin—particularly at the 100 mg/kg dose—significantly reduced this effect. In addition, a non-linear dose–response pattern was observed; that is, the low dose was effective, whereas higher doses did not provide the same level of protection.

**Fig. 2:**
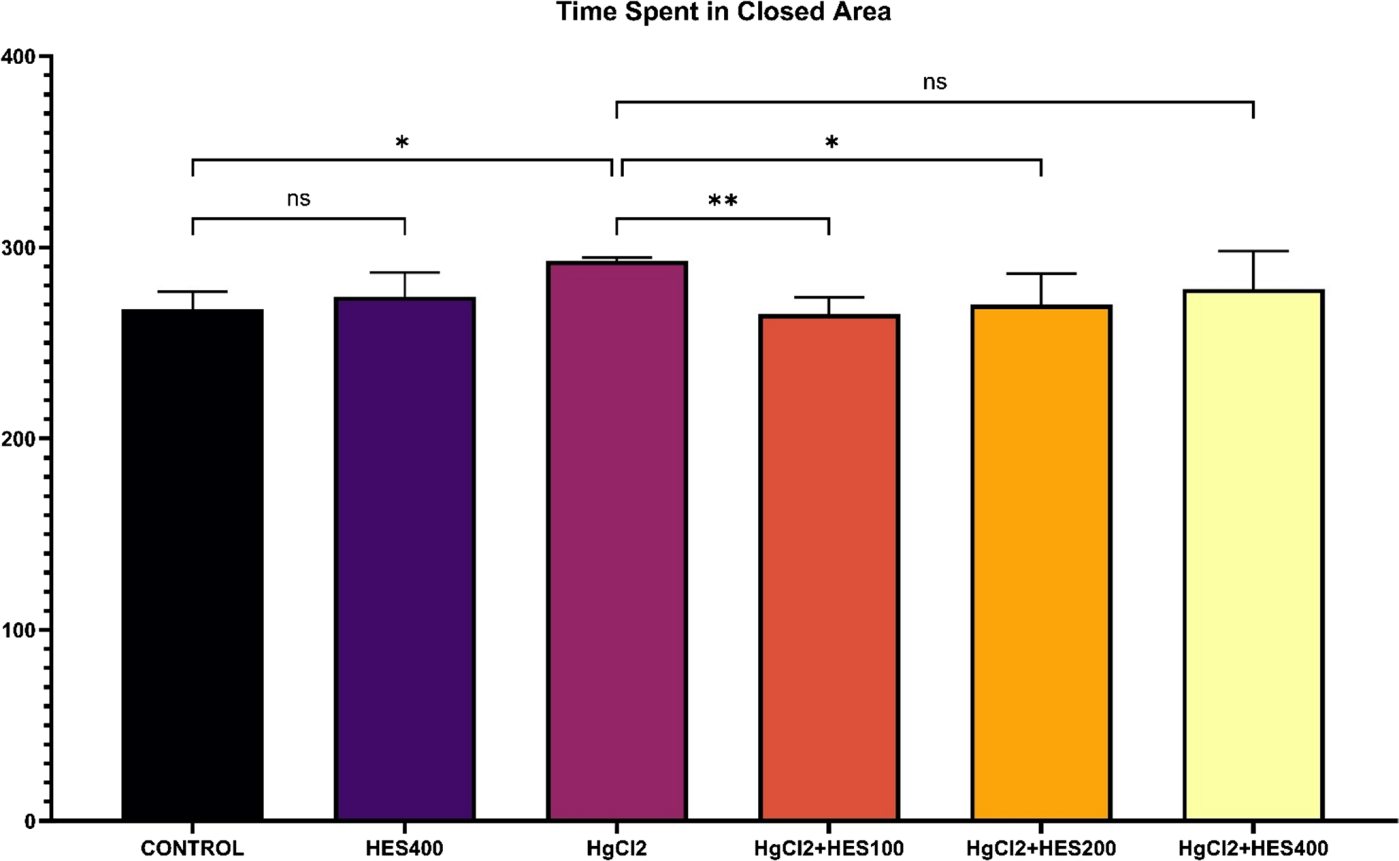
Statistical analysis graphs of the Elevated Plus Maze test (NS: *p* > 0.5, *p < 0.05, **p < 0.01, ***p < 0.001, ****p < 0.0001, *n* = 10) (One-Way ANOVA with Tukey’s post hoc test), GraphPad 10.6.1

The statistical findings show that the HgCl₂ group differed significantly from both the control and HES400 groups (*p* < 0.05), and that the most pronounced difference occurred between HgCl₂ and HgCl₂+HES100 (*p* < 0.01). No other comparisons reached statistical significance (ns). Therefore, the EPM test results indicate that hesperidin may exert a protective effect against anxiety-like behaviors, particularly at specific doses, and that this effectiveness varies depending on the dose administered (Fig. 2).

### Locomotor Activity Test

Locomotor activity analysis revealed that HgCl₂ markedly disrupted behavioral performance across all measured parameters. Stereotypic movements were significantly reduced in the HgCl₂ group compared with the control and HES400 groups (*p* < 0.0001). Co-administration of hesperidin (100, 200, and 400 mg/kg) significantly increased stereotypic behavior relative to HgCl₂ alone (*p* < 0.001).

Ambulatory activity was also diminished following HgCl₂ exposure (*p* < 0.05–0.01), whereas hesperidin co-treatment restored locomotor performance in a dose-dependent manner, with the HgCl₂+HES400 group showing values comparable to controls (*p* < 0.0001).

**Fig. 3:**
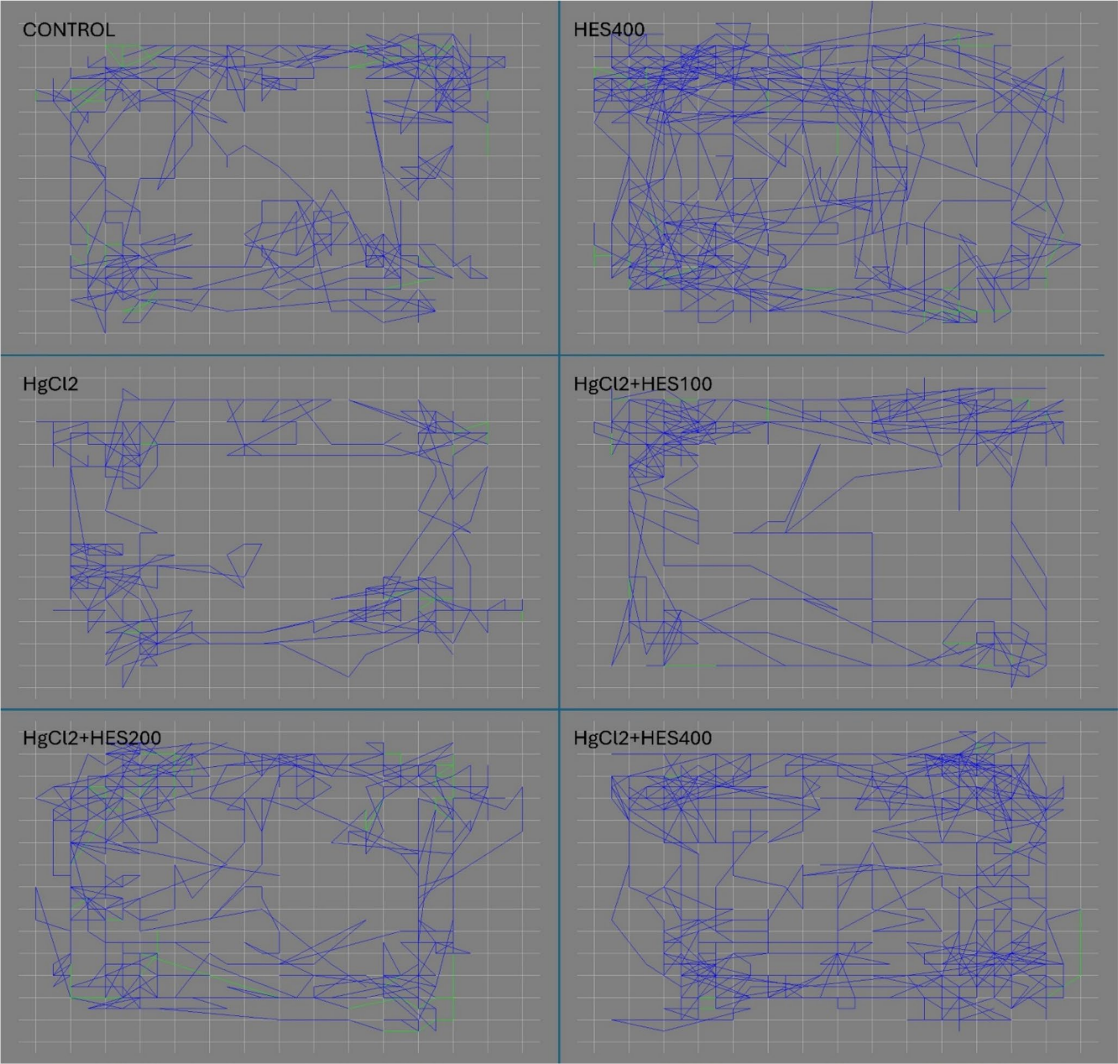
Representative infrared tracking map for each group in the locomotor activity test (green = vertical movements, blue = horizontal movements, *n* = 10)

Similarly, vertical activity (rearing count) was significantly suppressed in HgCl₂-treated rats (*p* < 0.01). Hesperidin increased rearing behavior in all co-treatment groups, although this improvement remained partial (Fig. 3).

In horizontal activity, a reduction was detected in the HgCl₂ group (*p* < 0.05), and activity was also lower in the HgCl₂+HES100 group; although a recovery trend was observed at higher doses (200–400 mg/kg), most comparisons did not reach statistical significance (ns). When the resting percentage was examined, a marked increase was observed in the HgCl₂ group (*p* < 0.05, *p* < 0.01), indicating elevated motor inhibition. In the hesperidin-treated groups, the resting percentage decreased significantly (*p* < 0.01) and approached control levels.

For the total distance parameter, a significant reduction was identified in the HgCl₂ group compared with the control and HES400 groups (*p* < 0.05). With hesperidin co-administration, total distance showed a recovery trend, and in the HgCl₂+HES100 group this increase reached statistical significance (*p* < 0.05). Overall, HgCl₂ suppressed stereotypic and exploratory behaviors, reduced horizontal movements, increased resting time, and decreased total distance. Hesperidin significantly improved most of these impairments—particularly at the low dose (100 mg/kg)—while providing partial but statistically non-significant recovery in certain parameters (such as horizontal activity).

**Fig. 4:**
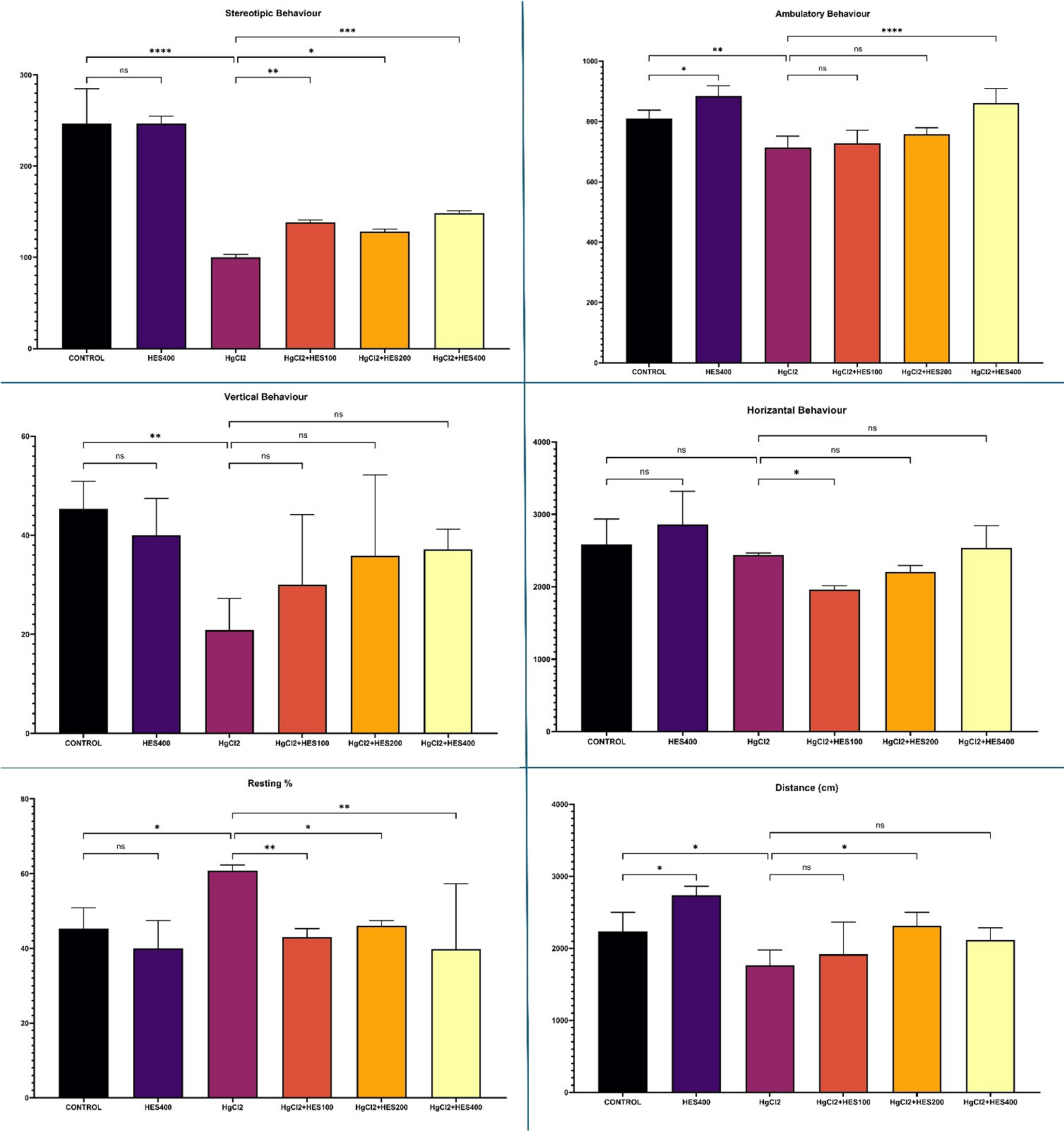
Statistical analysis graphs of the locomotor activity test (ns: *p* > 0.5, *p < 0.05, **p < 0.01, ***p < 0.001, ****p < 0.0001, *n* = 10) (One-Way ANOVA with Tukey’s post hoc test), GraphPad 10.6.1

Taken together, these findings demonstrate that HgCl₂ severely disrupts locomotor and behavioral capacity in rats, whereas hesperidin exhibits neuroprotective effects by ameliorating these deficits, especially at lower doses (Fig. 4). 

### Regulatory Effects of Hesperidin on MDA, SOD, and GSH in the Mercury Chloride-Induced Oxidative Stress Model

The study demonstrated that mercury chloride (HgCl₂) administration caused marked alterations in oxidative stress markers (MDA, SOD, and GSH), while hesperidin treatment ameliorated these adverse effects in a dose-dependent manner.

**Fig. 5:**
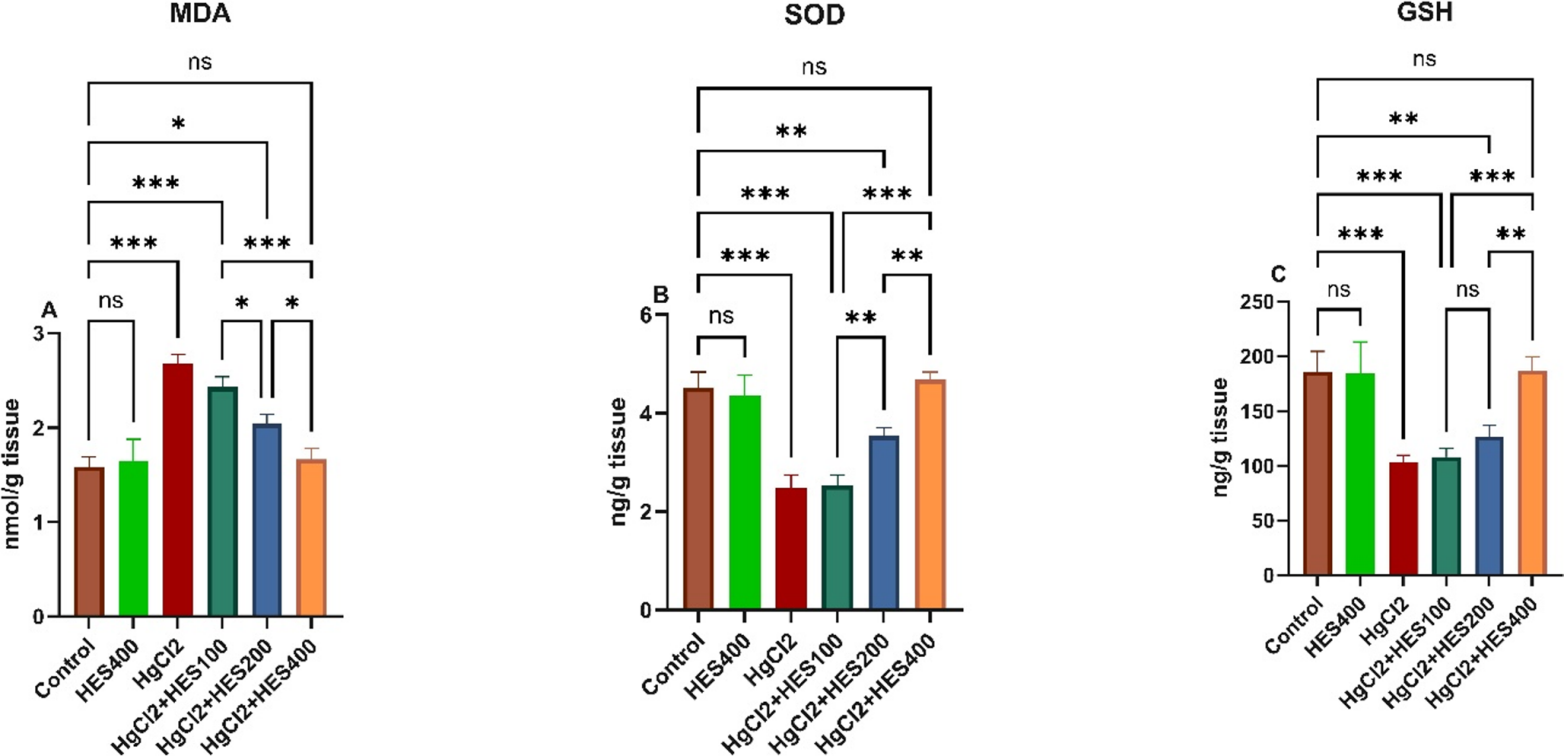
Distribution of MDA(**A**), SOD(**B**), and GSH(**C**) levels across experimental groups. Data are presented as mean ± SD. Statistical analysis was performed using one-way ANOVA followed by Tukey’s post hoc test. ***p < 0.001, **p < 0.01, *p < 0.05, *p* > 0.05, *n* = 10

HgCl₂ administration significantly increased MDA levels compared with the control group (*p* < 0.001), indicating a strong induction of lipid peroxidation by mercury exposure. Hesperidin treatment reduced MDA levels in a dose-dependent manner. In the HgCl₂ + HES100 group, a decrease in MDA was observed; however, the values remained higher than those of the control group (*p* < 0.001). In the HgCl₂ + HES200 group, the reduction became more pronounced (*p* < 0.05), and in the HgCl₂ + HES400 group, MDA levels approached control values, with no statistically significant difference between the two (*p* > 0.05, Fig. 5). Post hoc analysis further revealed that MDA levels in the HgCl₂ + HES400 group were significantly lower than those observed in the HgCl₂ + HES100 (*p* < 0.001) and HgCl₂ + HES200 groups (*p* < 0.05), indicating a superior protective effect at the highest dose (Fig. 5).

HgCl₂ administration led to a significant reduction in both SOD and GSH levels compared with the control group (*p* < 0.001). This decrease indicates suppression of the antioxidant defense system, which plays a crucial role in combating oxidative stress, and reflects a disruption of cellular redox balance. Hesperidin treatment produced dose-dependent improvements in both parameters. In the HgCl₂ + HES100 group, SOD and GSH levels remained low (*p* < 0.001), whereas in the HgCl₂ + HES200 group, a significant recovery was observed in both markers (*p* < 0.01). With high-dose hesperidin (HgCl₂ + HES400), SOD and GSH levels reached values comparable to those of the control group, with no statistically significant differences detected (*p* > 0.05, Fig. 5). GSH and SOD levels in the HgCl₂ + HES400 group were significantly higher than those in the HgCl₂ + HES100 group (*p* < 0.001), while a significant difference was also observed when compared with the HgCl₂ + HES200 group (*p* < 0.01) (Fig. 5).

### Regulatory Effects of Hesperidin on the Inflammatory Response Induced by HgCl₂ (TNF-α, IL-1β, IL-6, IL-10, NF-κB, and TLR4)

In this study, HgCl₂ exposure was found to increase pro-inflammatory cytokines (TNF-α, IL-1β, IL-6) and inflammatory signaling pathways (NF-κB, TLR4), while reducing the level of the anti-inflammatory cytokine IL-10. Hesperidin treatment corrected these alterations in a dose-dependent manner.

**Fig. 6:**
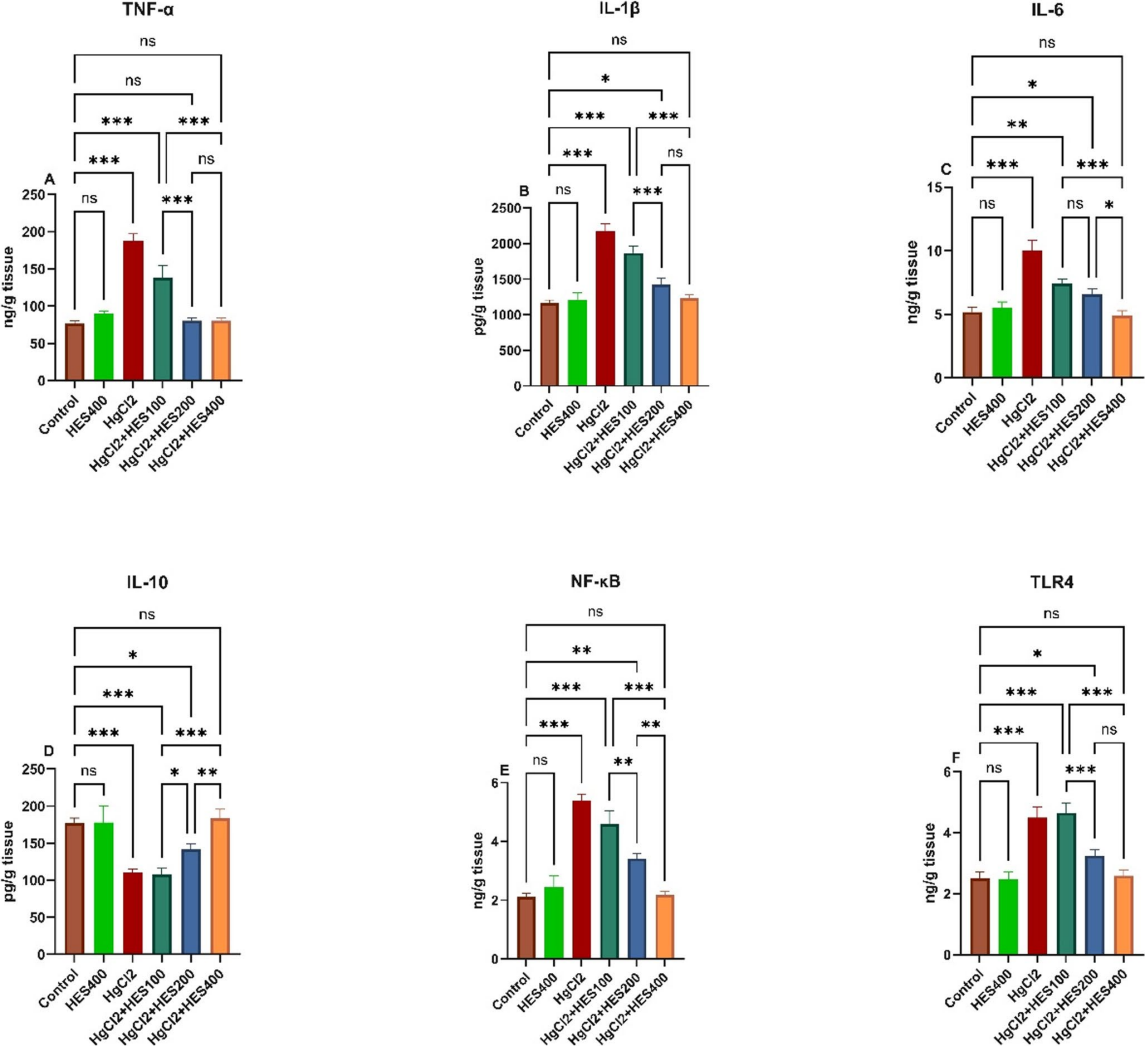
Distribution of TNF-α (**A**), IL-1β(**B**), IL-6(**C**), IL-10(**D**), NF-κB(**E**), and TLR4(**F**) levels across the groups. Data are presented as mean ± SD. Statistical analysis was performed using one-way ANOVA followed by Tukey’s post hoc test. ***p < 0.001, **p < 0.01, *p < 0.05, *p* > 0.05, *n* = 10

HgCl₂ administration led to marked increases in TNF-α, IL-1β, and IL-6 levels compared with the control group (*p* < 0.001), indicating a strong pro-inflammatory response induced by mercury. Hesperidin treatment suppressed these cytokines in a dose-dependent manner. In the HgCl₂+HES100 group, TNF-α and IL-1β levels remained elevated (*p* < 0.001), while IL-6 levels showed significant decreases (*p* < 0.01). In the HgCl₂+HES200 group, all parameters showed a more pronounced decrease, with IL-1β and IL-6 both reaching significance at the *p* < 0.05 level. High-dose hesperidin (HgCl₂+HES400) reduced TNF-α, IL-1β, and IL-6 levels to values very close to those of the control group, with no statistically significant differences detected (*p* > 0.05, Fig. 6). TNF-α, IL-1β, and IL-6 levels in the HgCl₂ + HES400 group were significantly lower than those in the HgCl₂ + HES100 group (*p* < 0.001). When compared with the HgCl₂ + HES200 group, TNF-α and IL-1β levels did not differ significantly (*p* > 0.05), whereas IL-6 levels were significantly lower (*p* < 0.05).

Anti-inflammatory cytokine IL-10 levels were significantly reduced by HgCl₂ administration compared with the control group (*p* < 0.001), demonstrating that mercury toxicity weakens inflammation-suppressing mechanisms. Hesperidin treatment increased IL-10 levels in a dose-dependent manner. No significant improvement was observed in the HgCl₂+HES100 group, where IL-10 levels remained low (*p* < 0.001), whereas the HgCl₂+HES200 group showed a marked elevation (*p* < 0.05). In the HgCl₂+HES400 group, IL-10 levels reached values similar to the control group, and no significant difference was recorded (*p* > 0.05, Fig. 6). IL-10 levels in the HgCl₂ + HES400 group were significantly different compared with the HgCl₂ + HES100 group (*p* < 0.001) and also showed a significant difference when compared with the HgCl₂ + HES200 group (*p* < 0.01).

HgCl₂ exposure significantly increased NF-κB and TLR4 levels compared with the control group (*p* < 0.001), both of which play key roles in initiating inflammatory responses. Hesperidin treatment dose-dependently suppressed these elevations. In the HgCl₂+HES100 group, NF-κB and TLR4 levels remained high (*p* < 0.001). In the HgCl₂+HES200 group, NF-κB showed a significant reduction (*p* < 0.01), and TLR4 decreased significantly (*p* < 0.05). In the HgCl₂+HES400 group, both parameters reached values very similar to the control group, with no statistically significant differences observed (*p* > 0.05, Fig. 6). In the HgCl₂ + HES400 group, NF-κB and TLR4 levels showed a significant difference compared with the HgCl₂ + HES100 group (*p* < 0.001). When compared with the HgCl₂ + HES200 group, NF-κB levels were significantly different (*p* < 0.01), whereas no significant difference was observed in TLR4 levels (*p* > 0.05).

### Histopathological Findings

**Fig. 7:**
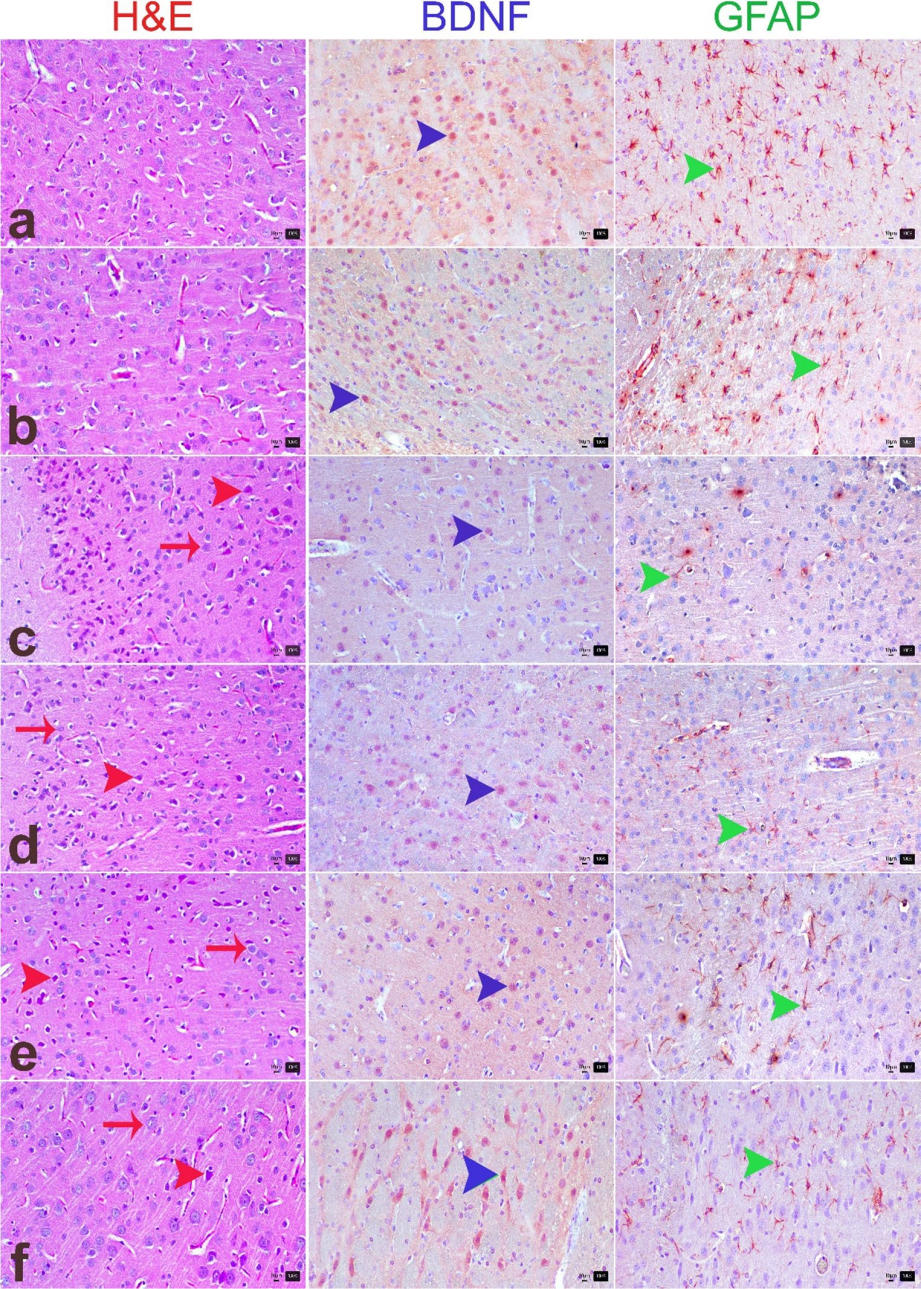
H&E and IHC staining images of the cerebral cortex region of brain tissues. The control group is shown in row (**a**), the HES400 group in row (**b**), the HgCl₂ group in row (**c**), the HgCl₂ + HES100 group in row (**d**), the HgCl₂ + HES200 group in row (**e**), and the HgCl₂ + HES400 group in row (**f**). In H&E staining, neuronal degeneration is indicated by red arrows and necrosis by red arrowheads. In IHC staining, neuronal BDNF expression is indicated by blue arrowheads, while astrocytic GFAP expression is indicated by green arrowheads. Staining methods: H&E and IHC-P. Objective: 20X, Scale bar: 10 μm, Zoom: 100%, *n* = 10

Histopathological examination of cerebral cortex tissue samples revealed that neurons in the control and HES400 groups exhibited normal morphology. In contrast, the HgCl₂ group showed severe degenerative and necrotic changes in neuronal cells. In the HgCl₂ + HES100 group, these pathological findings were reduced, although the improvement did not reach statistical significance (*p* > 0.05). In the HgCl₂ + HES200 and HgCl₂ + HES400 groups, the degenerative changes were significantly decreased compared with the HgCl₂ group (*p* < 0.05) (Fig. 7).

### Immunohistochemical and Immunofluorescence Findings

**Fig. 8:**
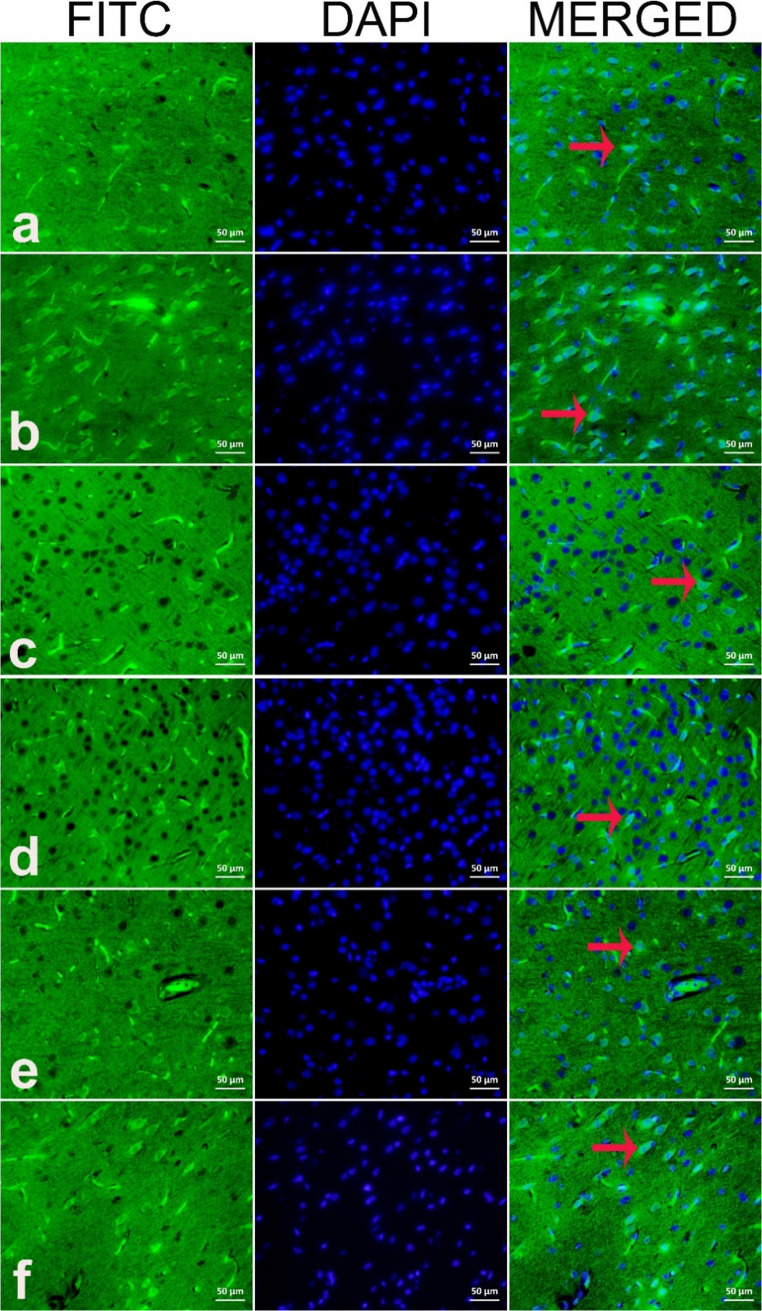
IF staining images of the cerebral cortex region of brain tissues. The control group is shown in row (**a**), the HES400 group in row (**b**), the HgCl₂ group in row (**c**), the HgCl₂ + HES100 group in row (**d**), the HgCl₂ + HES200 group in row (**e**), and the HgCl₂ + HES400 group in row (**f**). In the IF staining, neuronal Bcl-2 expression is indicated by red arrows. Staining method: IF. Objective: 40X, Scale bar: 50 μm. FITC: Secondary antibody, DAPI: Nuclear stain, MERGED: Combined visualization of FITC and DAPI, *n* = 10

**Fig. 9:**
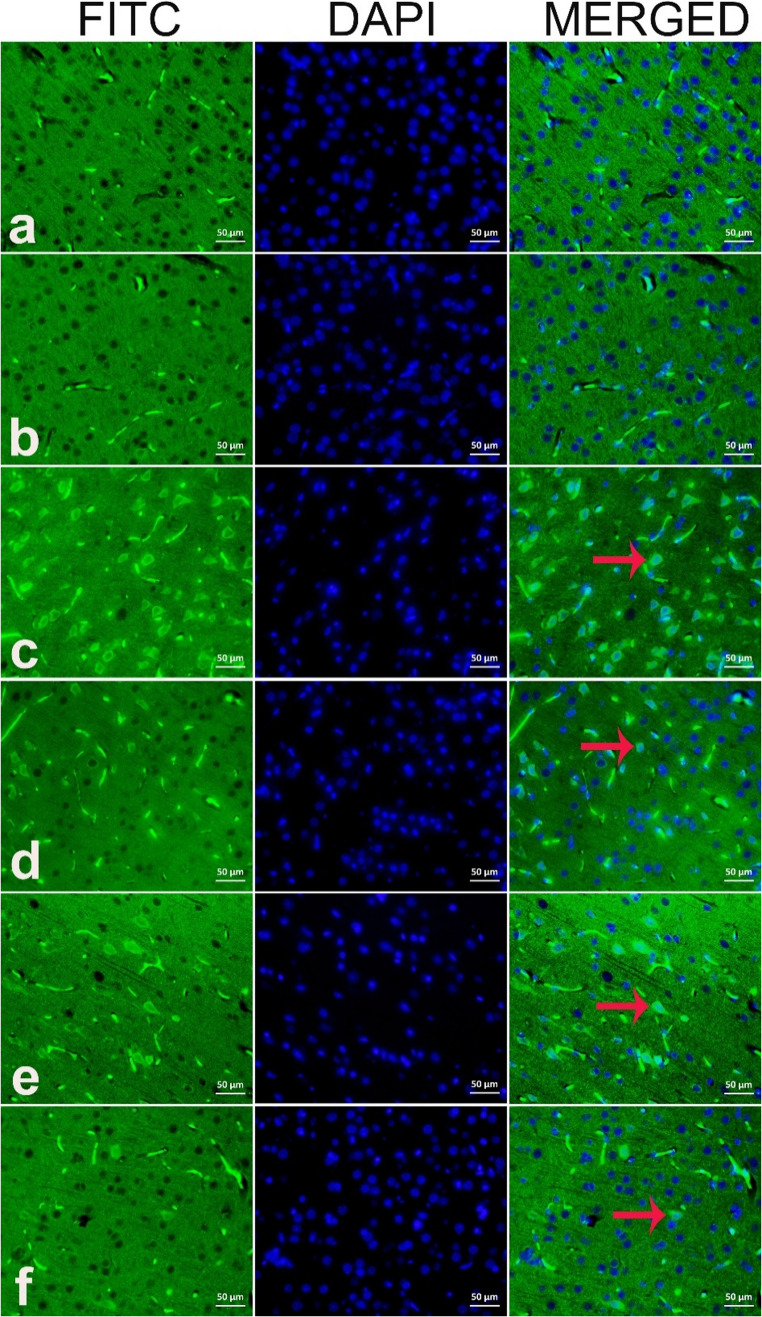
IF staining images of the cerebral cortex region of brain tissues. The control group is shown in row (**a**), the HES400 group in row (**b**), the HgCl₂ group in row (**c**), the HgCl₂ + HES100 group in row (**d**), the HgCl₂ + HES200 group in row (**e**), and the HgCl₂ + HES400 group in row (**f**). In IF staining, neuronal BAX expression is indicated by red arrows. Staining method: IF. Objective: 40X, Scale bar: 50 μm. FITC: Secondary antibody, DAPI: Nuclear stain, MERGED: Combined visualization of FITC and DAPI, *n* = 10

**Fig. 10:**
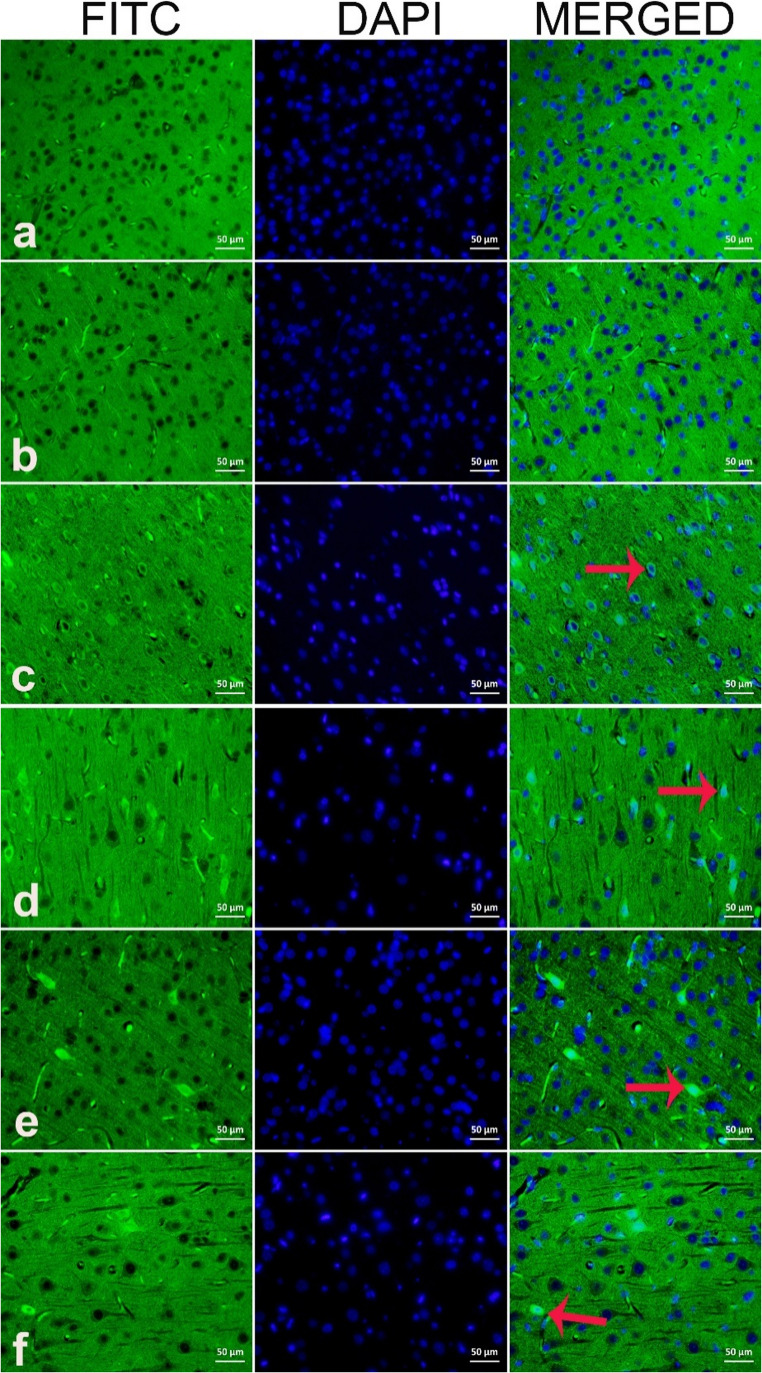
IF staining images of the cerebral cortex region of brain tissues. The control group is shown in row (**a**), the HES400 group in row (**b**), the HgCl₂ group in row (**c**), the HgCl₂ + HES100 group in row (**d**), the HgCl₂ + HES200 group in row (**e**), and the HgCl₂ + HES400 group in row (**f**). In IF staining, neuronal Caspase-3 expression is indicated by red arrows. Staining method: IF. Objective: 40X, Scale bar: 50 μm. FITC: Secondary antibody, DAPI: Nuclear stain, MERGED: Combined visualization of FITC and DAPI, *n* = 10

**Fig. 11:**
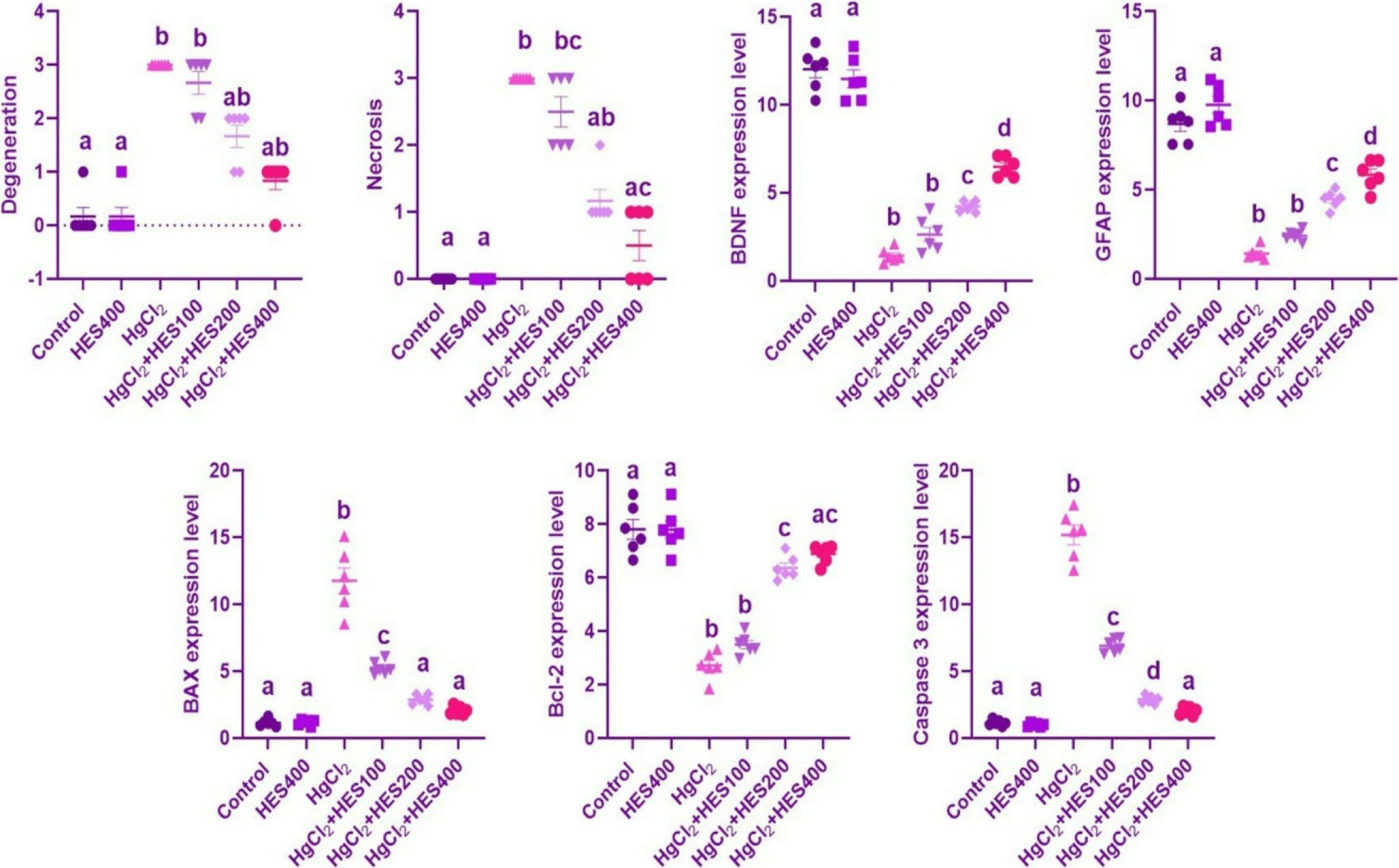
Histopathological, immunohistochemical, and immunofluorescence scoring data and their statistical analysis results are presented. For histopathological evaluations (degeneration and necrosis), statistical analyses were performed using the Kruskal–Wallis test followed by Dunn’s post hoc test. For IHC and IF staining, values obtained through ImageJ analysis were evaluated using One-Way ANOVA followed by Tukey’s post hoc test. In the graphs, letters differing from the control group “a” (such as b, c, or d) indicate statistically significant differences (*p* < 0.05). The Y-axis represents the scoring of findings, while the X-axis indicates the experimental groups. Data are expressed as mean ± SD, *n* = 10

In the IHC and IF staining of cerebral cortex tissue samples, strong expression levels of BDNF (Fig. 7), GFAP (Fig. 7), and Bcl-2 (Fig. 8) were observed in the control and HES400 groups, while low levels of BAX (Fig. 9) and Caspase-3 (Fig. 10) expression were detected. In the HgCl₂ group, BDNF, GFAP, and Bcl-2 expression levels were significantly reduced (*p* < 0.05) compared with the control and HES400 groups, whereas BAX and Caspase-3 levels were significantly increased (*p* < 0.05). In the treatment groups, these expression patterns approached those of the control and HES400 groups in a dose-dependent manner. The scores obtained from histopathological, immunohistochemical, and immunofluorescence evaluations, along with their statistical analyses, are presented in Fig. 11.

### Protective Effect of Hesperidin against HgCl₂-Induced Endoplasmic Reticulum Stress

To evaluate endoplasmic reticulum (ER) stress–related alterations in protein expression within cerebral cortex tissue, CHOP, XBP1, GRP78, PERK, ATF4, and IRE1 proteins were examined by Western blot analysis. The effects of different doses of hesperidin (100, 200, 400 mg/kg) were compared against the stress response induced by HgCl₂ exposure. Protein levels were normalized to β-tubulin and analyzed densitometrically.

**Fig. 12:**
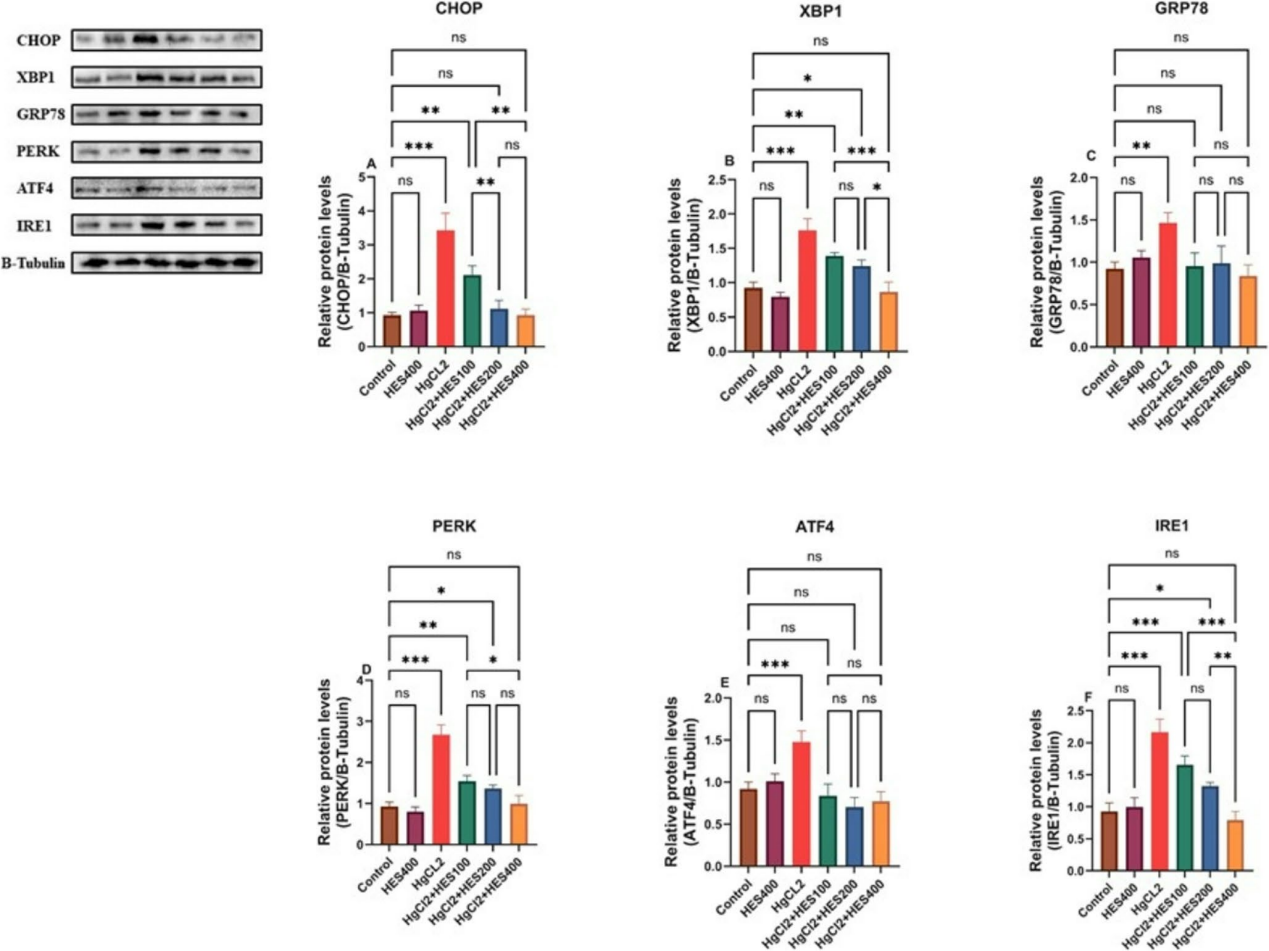
Distribution of CHOP(**A**), XBP1(**B**), GRP78(**C**), PERK(**D**), ATF4(**E**), and IRE1(F) protein expression levels across the groups. Data are presented as mean ± SD. Statistical analysis was performed using one-way ANOVA followed by Tukey’s post hoc test. ***p < 0.001, **p < 0.01, *p < 0.05, *p* > 0.05, *n* = 10

CHOP, XBP1, and GRP78 protein expression levels were significantly increased in the HgCl₂-treated group compared with the control (CHOP *p* < 0.001; XBP1 *p* < 0.001; GRP78 *p* < 0.01). Hesperidin administration reduced these elevations in a dose-dependent manner; in the HgCl₂+HES100 group, significant decreases were observed for CHOP (*p* < 0.01) and XBP1 (*p* < 0.01). GRP78 expression in the HgCl₂+HES100 group did not show a significant difference compared with the control (*p* > 0.05). In the HgCl₂+HES200 group, CHOP (*p* > 0.05), XBP1 (*p* < 0.05), and GRP78 (*p* > 0.05) levels approached control values. In the HgCl₂+HES400 group, CHOP, XBP1, and GRP78 expression levels were not significantly different from the control group (*p* > 0.05). When compared with the HgCl₂ + HES100 group, the HgCl₂ + HES400 group showed statistically significant differences in CHOP (*p* < 0.01) and XBP1 levels (*p* < 0.001). In comparison with the HgCl₂ + HES200 group, a significant difference was observed in XBP1 levels (*p* < 0.05, Fig. 12A, B and C).

PERK, ATF4, and IRE1 protein expression levels were markedly increased in the HgCl₂ group compared with the control (PERK *p* < 0.001; ATF4 *p* < 0.001; IRE1 *p* < 0.001). In the HgCl₂+HES100 group, PERK levels showed a significant reduction (*p* < 0.01), whereas ATF4 levels did not exhibit a significant change and were close to control values (*p* > 0.05). For IRE1, a significant difference was observed in the HgCl₂+HES100 group (*p* < 0.001), indicating that its levels remained significantly altered compared with the control. In the HgCl₂+HES200 group, both PERK (*p* < 0.05) and IRE1 (*p* < 0.05) levels showed significant decreases. ATF4 levels in this group did not show a significant difference compared with the control (*p* > 0.05). In the HgCl₂+HES400 group, PERK, ATF4, and IRE1 expression levels were close to control values, and no statistically significant differences were detected among the groups (*p* > 0.05). When compared with the HgCl₂ + HES100 group, the HgCl₂ + HES400 group showed significant differences in PERK (*p* < 0.05) and IRE1 levels (*p* < 0.001). In comparison with the HgCl₂ + HES200 group, IRE1 levels in the HgCl₂ + HES400 group were significantly lower (*p* < 0.01, Fig. 12D, E and F).

### Effects of Hesperidin on HgCl₂-Induced Autophagic Response (Beclin-1, LC3A, LC3B) at the mRNA Expression Level

To determine the activation level of the autophagic process, the relative expression levels of the autophagy-related genes Beclin-1, LC3A, and LC3B were evaluated.

**Fig. 13:**
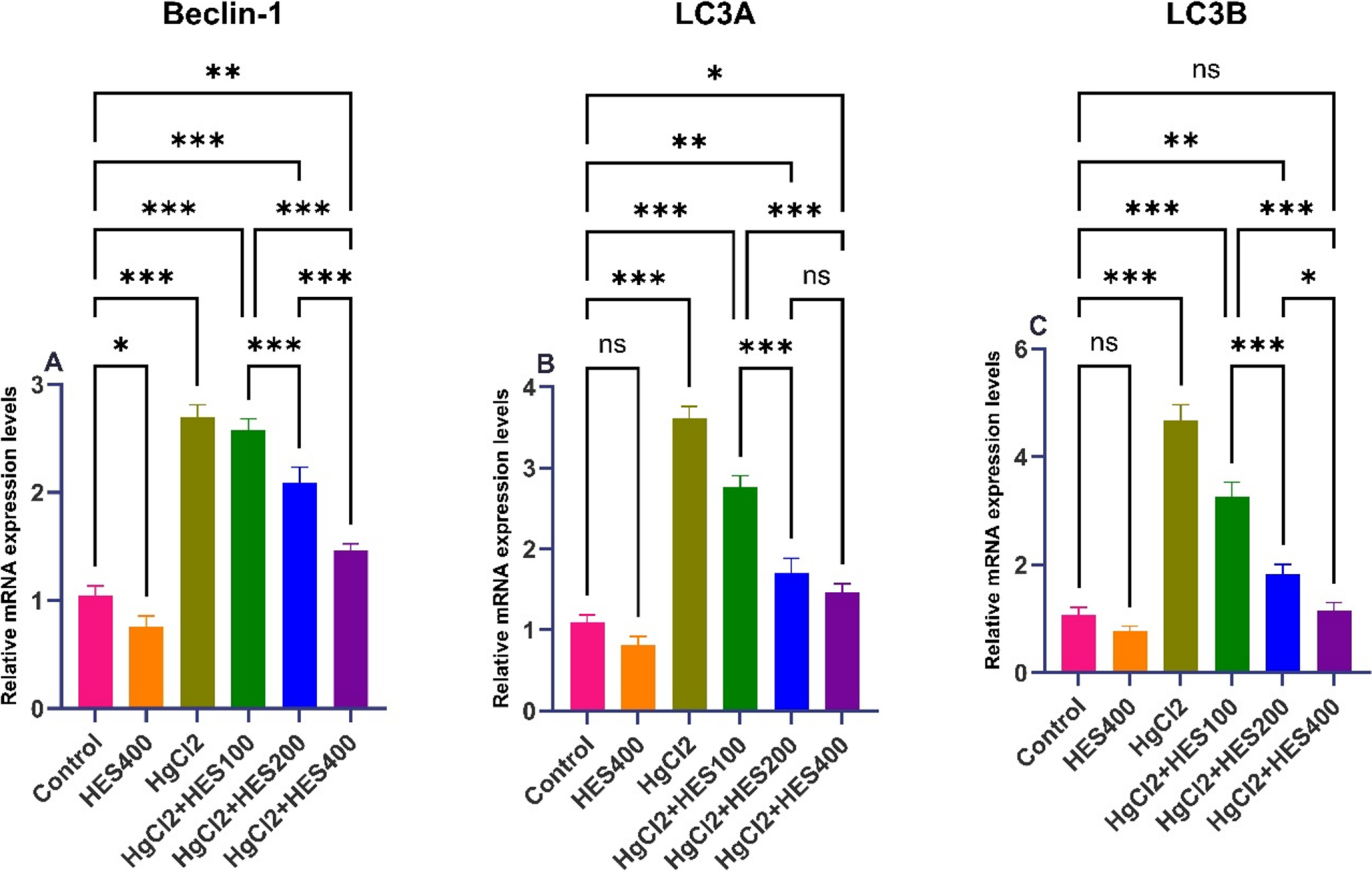
Distribution of Beclin-1(**A**), LC3A(**B**), and LC3B(**C**) gene expression levels across the groups. Data are presented as mean ± SD. Statistical analysis was performed using one-way ANOVA followed by Tukey’s post hoc test. ***p < 0.001, **p < 0.01, *p < 0.05, *p* > 0.05, *n* = 10

HgCl₂ administration led to significant increases in the mRNA expression levels of Beclin-1, LC3A, and LC3B compared with the control group (*p* < 0.001). In the HgCl₂+HES100 group, all three autophagy markers remained elevated, with increases in Beclin-1, LC3A, and LC3B reaching statistical significance at the *p* < 0.001 level. In the HgCl₂+HES200 group, Beclin-1 expression differed significantly from the control group at the *p* < 0.001 level, while LC3A and LC3B expression levels differed at the *p* < 0.01 level. In the HgCl₂+HES400 group, Beclin-1 expression was significantly different from the control (*p* < 0.01), and LC3A expression showed a significant difference at the *p* < 0.05 level. In contrast, LC3B levels in this group were close to control values, with no statistically significant difference observed (*p* > 0.05). Additionally, in the HES400 group, Beclin-1 levels were lower than those of the control group (*p* < 0.05), while LC3A and LC3B levels were similar to the control (Fig. 13). When the HgCl₂ + HES400 group was compared with the HgCl₂ + HES100 group, significant differences were observed in Beclin-1, LC3A, and LC3B levels (*p* < 0.001). In comparison with the HgCl₂ + HES200 group, Beclin-1 levels showed a significant difference (*p* < 0.001), while LC3B levels were also significantly different (*p* < 0.05, Fig. 13).

## Discussion

Mercury chloride (HgCl₂) is a well-recognized environmental toxicant that exerts its neurotoxic effects through a complex and interconnected network of molecular disturbances, including oxidative stress, inflammation, mitochondrial dysfunction, apoptosis, autophagy, and endoplasmic reticulum (ER) stress [5, 22, 23]. These mechanisms interact in a nonlinear manner and collectively contribute to synaptic dysfunction, impaired neurotrophic support, glial reactivity, and ultimately neurodegeneration [24, 25, 26, 27]. Growing evidence suggests that natural flavonoids, particularly hesperidin, possess broad-spectrum protective effects due to their multimodal antioxidant, anti-inflammatory, and cytoprotective properties [28, 29, 30, 31, 32]. In this context, the present study demonstrates that hesperidin provides multi-level neuroprotection against HgCl₂-induced injury by modulating key pathological pathways and restoring molecular, cellular, and behavioral integrity.

Behaviorally, HgCl₂ exposure significantly increased anxiety-like responses and impaired locomotor activity. Interestingly, hesperidin exhibited a non-linear dose–response, with the 100 mg/kg dose showing notable efficacy in EPM parameters, while higher doses more strongly influenced oxidative and inflammatory markers. The non-linear dose–response pattern observed in the EPM test may reflect differential sensitivity of behavioral endpoints compared with molecular pathways. Lower doses of hesperidin may be sufficient to modulate anxiety-related behaviors, whereas higher doses may preferentially exert effects at the molecular and cellular levels without producing proportional behavioral changes. Such non-linear patterns have been reported in other toxicological models, where flavonoids exert optimal behavioral effects at lower doses while higher doses more prominently regulate molecular pathways [33, 34]. The improvements in locomotor and anxiety-related behaviors observed in our study correlate with the restoration of BDNF levels and reduced glial activation, suggesting that hesperidin’s behavioral benefits may be partly mediated through the recovery of neurotrophic signaling and astrocytic homeostasis.

Oxidative stress represents one of the earliest and most dominant consequences of HgCl₂ neurotoxicity. Consistent with previous findings, we observed marked increases in MDA levels and reductions in SOD and GSH activities, indicating significant impairment of endogenous antioxidant defenses [23, 24, 25, 35]. Hesperidin, particularly at 400 mg/kg, effectively restored these parameters. This aligns with extensive literature showing that hesperidin enhances antioxidant capacity, stabilizes redox homeostasis, and prevents oxidative membrane damage through direct ROS scavenging and upregulation of endogenous antioxidants [29, 30, 36, 37]. Since oxidative stress amplifies downstream pathways such as mitochondrial dysfunction, apoptosis, and ER stress, the robust antioxidant response induced by hesperidin likely played a central role in the broad protective profile observed in this study.

Inflammatory activation constitutes another major mechanism in HgCl₂-induced neurotoxicity. Our findings showed elevated TNF-α, IL-1β, and IL-6 levels and suppressed IL-10 in the toxic group, consistent with earlier reports indicating that heavy metals activate microglia, enhance NF-κB signaling, and disrupt cytokine balance [5, 22, 38]. Hesperidin significantly reduced pro-inflammatory cytokines and restored IL-10 levels, in agreement with studies demonstrating that flavonoids inhibit Toll-like receptor signaling, reduce NF-κB activation, and prevent glial reactivity [5, 12, 28, 35, 39, 40]. The normalization of cytokine balance observed here likely contributed to the preservation of neuronal morphology and the improvement in behavioral performance.

Histopathological examinations revealed substantial neuronal degeneration and necrosis in the HgCl₂ group, confirming previous findings of heavy-metal-induced cortical atrophy, synaptic deterioration, and neuronal shrinkage[28, 41]. Hesperidin treatment—especially at 200 and 400 mg/kg—significantly reduced these pathological alterations, supporting its neuroprotective role through antioxidant and anti-inflammatory mechanisms. This is consistent with earlier studies showing that hesperidin protects neural structure in various models of neurotoxicity by stabilizing cellular membranes and preventing excessive excitotoxic and inflammatory damage [14, 42].

Apoptotic dysregulation is a key contributor to mercury-induced neuronal injury. The increased Bax and caspase-3 expression and reduced Bcl-2 levels observed in our study align with previous reports of mercury-induced mitochondrial destabilization and caspase activation [4, 5, 22, 28]. Hesperidin significantly reversed these changes, demonstrating strong anti-apoptotic activity. These results are supported by findings from Küçükler [32], Khezri [30], and Kumar [37], who reported that hesperidin modulates intrinsic apoptotic pathways by reducing ROS-driven mitochondrial dysfunction and restoring anti-apoptotic proteins [12, 29, 30].

Neurotrophic factor disruption and glial activation represent additional pathological consequences of mercury exposure. The reduced BDNF levels observed in the HgCl₂ group are consistent with diminished synaptic plasticity and impaired neuronal resilience [23, 24]. Meanwhile, elevated GFAP expression indicates astrocytic activation and progression of neuroinflammation [12, 30]. Hesperidin dose-dependently restored BDNF and strongly suppressed GFAP expression, suggesting that it improves both neuronal support systems and glial homeostasis [29, 43]. Restoration of BDNF is particularly important, as it enhances synaptic recovery, promotes neuronal survival, and may underlie the behavioral improvements observed in this study.

ER stress and unfolded protein response (UPR) activation constitute another major mechanism of mercury-induced neurotoxicity. Consistent with Rana [47] and Demircan [46], we observed significant elevations in GRP78, PERK, ATF4, XBP1, and CHOP, indicative of UPR activation and pro-apoptotic ER stress signaling [44, 45, 46, 47]. Hesperidin robustly reduced these markers, consistent with evidence that flavonoids attenuate PERK/ATF4/CHOP-mediated ER dysfunction and protect neurons from misfolded-protein–associated apoptosis [36, 39, 48]. The strong modulation of ER stress observed here highlights hesperidin’s potential to target deep cellular stress pathways not typically addressed by classical antioxidants.

Autophagic dysregulation also contributes to mercury-induced neuronal injury. Elevated Beclin-1 and LC3A/B expression in the HgCl₂ group reflects pathological activation of autophagy [40, 49, 50]. Hesperidin attenuated these elevations, particularly at high doses, demonstrating its ability to prevent excessive autophagic signaling. This is consistent with Varışlı [52] and Kahkesh [51], who reported that hesperidin has a context-dependent regulatory role, enhancing autophagy when insufficient and suppressing it when excessive [51, 52]. This bidirectional modulation underscores hesperidin’s ability to restore autophagic balance under toxic stress conditions.

One limitation of the present study is that the hesperidin used was not of pharmaceutical-grade purity but rather reflects the commercially available formulation commonly used in experimental studies. Therefore, future investigations employing higher-purity formulations may provide more precise mechanistic insights and enhance translational relevance.

## Conclusion

Taken together, the results of this study demonstrate that hesperidin exerts broad neuroprotective effects against mercury chloride–induced neurotoxicity by modulating several key pathological pathways. By restoring redox homeostasis, reducing pro-inflammatory cytokine release, normalizing apoptotic protein expression, attenuating endoplasmic reticulum stress responses, and preventing excessive autophagic activation, hesperidin contributed to the preservation of neuronal integrity at molecular, cellular, and behavioral levels. Furthermore, its ability to enhance BDNF expression while suppressing GFAP-mediated astrocytic activation suggests a supportive role in maintaining synaptic plasticity and neural network stability. Overall, these findings indicate that hesperidin may have therapeutic potential in mitigating heavy metal–induced neuronal damage; however, further in vitro and in vivo studies are required to clarify its mechanisms of action and to validate its translational relevance in environmental neurotoxicant exposure and neurodegenerative conditions associated with oxidative and inflammatory stress.

## Data Availability

The datasets generated and/or analyzed during the current study are not publicly available due to institutional restrictions and the need to protect laboratory raw data integrity but are available from the corresponding author on reasonable request.
